# Assessing the Utility of a Quality-of-Care Assessment Tool Used in Assessing Comprehensive Care Services Provided by Community Health Workers in South Africa

**DOI:** 10.3389/fpubh.2022.868252

**Published:** 2022-05-16

**Authors:** Olukemi Babalola, Jane Goudge, Jonathan Levin, Celia Brown, Frances Griffiths

**Affiliations:** ^1^Centre for Health Policy, Faculty of Health Sciences, University of the Witwatersrand, Johannesburg, South Africa; ^2^Department of Epidemiology and Biostatistics, Faculty of Health Sciences, University of the Witwatersrand, Johannesburg, South Africa; ^3^Division of Health Sciences, University of Warwick, Warwick Medical School, Coventry, United Kingdom

**Keywords:** community health workers, quality-of-care assessment tool, utility, validity, reliability, comprehensive care

## Abstract

**Background:**

Few studies exist on the tools for assessing quality-of-care of community health worker (CHW) who provide comprehensive care, and for available tools, evidence on the utility is scanty. We aimed to assess the utility components of a previously-reported quality-of-care assessment tool developed for summative assessment in South Africa.

**Methods:**

In two provinces, we used ratings by 21 CHWs and three team leaders in two primary health care facilities per province regarding whether the tool covered everything that happens during their household visits and whether they were happy to be assessed using the tool (acceptability and face validity), to derive agreement index (≥85%, otherwise the tool had to be revised). A panel of six experts quantitatively validated 11 items of the tool (content validity). Content validity index (CVI), of individual items (I-CVI) or entire scale (S-CVI), should be >80% (excellent). For the inter-rater reliability (IRR), we determined agreement between paired observers' assigned quality-of-care messages and communication scores during 18 CHW household visits (nine households per site). Bland and Altman plots and multilevel model analysis, for clustered data, were used to assess IRR.

**Results:**

In all four CHW and team leader sites, agreement index was ≥85%, except for whether they were happy to be assessed using the tool, where it was <85% in one facility. The I-CVI of the 11 items in the tool ranged between 0.83 and 1.00. For the S-CVI, all six experts agreed on relevancy (universal agreement) in eight of 11 items (0.72) whereas the average of I-CVIs, was 0.95. The Bland-Altman plot limit of agreements between paired observes were −0.18 to 0.44 and −0.30 to 0.44 (messages score); and −0.22 to 0.45 and −0.28 to 0.40 (communication score). Multilevel modeling revealed an estimated reliability of 0.77 (messages score) and 0.14 (communication score).

**Conclusion:**

The quality-of-care assessment tool has a high face and content validity. IRR was substantial for quality-of-care messages but not for communication score. This suggests that the tool may only be useful in the formative assessment of CHWs. Such assessment can provide the basis for reflection and discussion on CHW performance and lead to change.

## Introduction

Community health workers (CHWs) are recruited from the community they serve, and after limited training, they provide community-based services (1–7). Many CHWs programs engaged in disease-specific interventions focusing on single diseases and conditions (family planning, antenatal care, and immunization services) (8, 9). Increasing evidence are suggesting that CHW programs are training CHWs to become generalists, providing more comprehensive healthcare (10, 11). These include maternal and child health, childhood immunization and breastfeeding, and diagnosis and treatment of illnesses (9). What is included as comprehensive services of CHW differ between countries and CHW programs (9–15). In South Africa, CHWs provide comprehensive promotive and preventive healthcare services, but do not treat illness (12–15). To enable effective implementation of CHW programs, we need to be able to assess the quality of comprehensive care provided by CHWs (16, 17).

There is a lack of validated tools, indicators, and standardized metrics to monitor CHW programs (9, 17–19). To guide the development of standardized measures of CHW performance, Agarwal, Sripad et al. proposed the “Community Health Worker Performance Measurement Framework” (20). This framework identifies “CHW knowledge”, “service delivery,” “service quality” and “data reporting” as indicators of CHW performance. Evidence from existing literature assessing CHW performance, shows that few of existing tools used are explicitly validated (see Supplementary Table 1 for existing literature categorized by the Agarwal Sripad et al. indicators). Several of these assessment methods target single diseases or conditions, or only assessed limited services, which is of limited use for assessing the quality-of-care of CHWs who provide comprehensive care. Laurenzi et al. piloted an assessment tool with some similarity to the tool we have assessed in our study. This Home Visit Communication Skills Inventory, a 21-item checklist, assessed only the communication skills (including domains on active listening, active delivery, and active connecting) of CHWs who provide comprehensive care, in South Africa (21). The Inventory scored audio-recorded and transcribed communication between the CHW and householder(s) during home visits. However, it does not assess CHW skills overall. Furthermore, global rating scales (22–28) used to assess a performance or skills (29) and which tend to be briefer than checklists (30) are also limited, because of concerns around inter-rater reliability (IRR) (31, 32).

We previously reported how we developed the quality-of-care assessment tool within a 3-year (2016–2019) intervention study (Batlhokomedi project), aimed to improve CHW quality-of-care in a South African district (33, 34). We designed the tool to undertake a summative assessment of CHW quality-of-care delivered during household visits (35–37). The tool was designed for an intervention study focused on the continuing assessment of CHWs during household visits taking into consideration the independent care provided by CHWs in the community and the several outcomes that are involved in comprehensive care (33, 34). Briefly, before setting out, just before and on entry, and during household visit, we assessed the various components of the CHW working day while on a household visit, with the tool structured according to the flow (Table 1) (33). Items in our quality-of-care assessment tool require a categorical response (e.g., yes/no; present/absent, scored as 1/0). The data scores obtained by direct observations are aggregated and through further calculations, are used to derive message and communication scores (separately) per household. The proportion of expected messages given, and actions undertaken per household, as well as the proportion of items with positive outcome while assessing communication are reported as the household message or communication score, respectively. By these methods, our tool can be used to derive easy-to-understand scores of performance domains. Finally, the CHW bag content and the conditions for which the CHW provides advice/messages and actions for the householders, are based on the South Africa national CHW training manual (38). Therefore, these are context specific. Trained non-clinical fieldworkers complete the tool while shadowing CHWs during household visits, guided by a fieldwork manual (33). It should be noted that in our tool, we only included items that the fieldworkers can easily grade and observe. Our tool could be used to measure the indicator subdomains (“CHW knowledge,” “service delivery,” “service quality” and “data reporting”) under the “community health systems performance output-CHW level” domain in the Agarwal, Sripad et al. performance framework (20).

**Table 1:** The quality-of-care assessment tool structure.

**Tool sections (point at which recorded)**	**Components of the tool**
Before setting out	Contents of CHW bag including the list of equipment available for use during the household visit
Just before and on entry to a household (CHW seeks for permission for the fieldworker to observe the visit)	Visit planning
	CHW communication skills including attention to confidentiality
During household visit	Householder conditions and messages and actions expected of CHW
	CHW communication skills including attention to confidentiality
After leaving the household	Factors that could prevent a CHW delivering good quality care
	CHW communication skills including attention to confidentiality

*CHW, community health worker*.

Our study aimed to assess the utility (face and content validity, acceptability, and inter-rater reliability) of the quality-of-care assessment tool developed to assess the quality of care of CHWs who perform comprehensive care during household visits. We sought to answer the question: Is the quality-of-care assessment tool valid or useful for assessing the quality of comprehensive care provided by CHWs during household visits?

## Methods

Assessment tools with high utility (reliability [whether an assessment result can be replicated assuming the same or similar conditions], validity [whether the items in an instrument of interest measure what it is intended to], educational impact [the extent to which desired educational goal expected of the learner and communicated through assessments are being achieved], cost and feasibility [evaluation of the balance between cost and benefit of implementing an instrument, and whether it is capable of being carried out successfully using available resources] and acceptability [extent to which relevant stakeholders consider the instrument acceptable as an assessment tool), are essential, to consider an assessment as ‘fair' (39). The extent to which these utility components are relevant depends on whether the assessment is formative, or summative (39). In this current study, we provide data on the utility (validity, acceptability, and inter-rater reliability) of an inventory, which covers communication, content and factors (CHW and household characteristics) likely to influence the ability of the CHW to provide good quality care (33, 34). Table 2 shows the study design, data collection methods and participants' selection criteria for each utility type.

**Table 2:** Type of utility, study design, data collection method, participants and data collected.

**Type of utility**	**Study design**	**Data collection method**	**Type of data**	**Number of participants**	**Selection criteria**	**Data collected**
Face validity and acceptability	Cross-sectional study using purposive sampling technique	Workshop, followed by self-completed questionnaire	Primary	6–10 per facility (four facilities)	Who could read fairly easily or were proficient or confident in the use of English. –Provided consent	-CHW and OTL years of experience –Ward-based Outreach Team Phases one and two examinations passed –Degree to which CHWs agree that the tool covers everything that happens during a household visit–Views of CHWs on the content of the tool –Degree to which CHWs agree that they will be happy for their home visits to be assessed using the tool. –Views of CHWs on why they would be happy for the tool to be used in assessing their performance
Content validity	Cross-sectional study using convenience sampling technique	Expert validation questionnaire	Primary	six	–At least a master's degree –Had worked on CHW issue for at least 3 years –Have at least three relevant publications or reports related to CHW performance within the South African context.	Experts' provided scores on 11 items in the tool
Inter-rater reliability	Primary data for this secondary data analysis were collected using mixed methods (including a cross-sectional observational study).	Direct observation by paired observers using the quality-of-care assessment tool	Secondary	three (household observations, *n =* 18)	Trained observers	- Observers' quality-of-care communication scores based on how the CHW engaged with the household -Observers' quality-of-care messages scores based on the health conditions identified and messages given by CHWs

*CHW, community health worker; OTL, outreach team leader*.

### Acceptability and Face Validity

For the acceptability and face validity, the CHWs and their team leaders (known as outreach team leaders [OTLs]), provided answers on the extent to which our tool covers everything that happens on household visits and how happy they were for their home visits to be assessed using the tool?” (40). That is, the CHWs who would be impacted by the assessment tool needed to express how they felt regarding the tool. Face validity can ensure acceptance of the tool and cooperation of the impacted clients and policy makers toward its use when non-professionals whose care will be assessed by the tool provide ratings on the tool wholly or its items (41) with or without further explanations regarding the ratings (42). The recommended face validity rating is on a five-point scale, from one “*the test is unsuitable for that purpose*” to five “*the test is extremely suitable for that purpose*.”

#### Study Design, Study Setting, and Participant Selection

Using a cross-sectional study design and purposive sampling technique, we collected primary data from CHWs and OTLs in two primary health care facilities each from Mpumalanga and Gauteng Provinces. To select each participant, in each facility, we asked the OTLs to (1) identify CHWs who met our selection criteria (see Table 2), and (2) who agreed to participate.

#### Data Collection Approaches

##### Workshop

Participants were asked to describe typical household visit day activities. Then, we checked whether the tool was describing these and discussed differences. We audio-recorded the workshop.

#### Self-Completed Questionnaire

We asked the participants to provide a score on a scale of one (*strongly disagree*) to five (*strongly agree*), for the following: (1) “*This quality-of-care assessment tool covers everything that happens on household visits*” and (2) “*I would be happy for my home visits to be assessed using this tool*.” We also asked the participants to provide qualitative comments regarding their views on the content of the tool and why they would be happy for the tool to be used in assessing their performance.

#### Data Analysis

From the CHWs/OTLs ratings, we calculated separately for the CHWs and OTLs the agreement index as the mean scores of ratings of the extent to which CHWs/OTLs agreed that the tool covers everything that happens on household visits (agreed) and how many were happy to be assessed using the tool (happy). The agreement index should be ≥85% (43) otherwise, the tool should be revised if the index is <85%.

### Content Validation

The content validity study requires that content experts determine the extent to which items in a tool are relevant or representative of intended constructs (44, 45). This a posteriori attempt to evaluate the relevance of the content of a scale, requires about 3–10 experts (46). A content validity index (CVI), which expresses the proportion of agreement on the assigned rating for relevancy of each item on a scale of zero and one, is calculated (46, 47). On a 4-point scale, experts' ratings of three or four is assigned ‘1' and one or two is assigned ‘0' (46). CVI assesses the relevancy of individual items (I-CVI) or the scale (S-CVI, i.e., for the tool as a whole) (46). As reported in previous studies (48, 49) we computed both the I-CVI as well as S-CVI (S-CVI/universal agreement [UA] and S-CVI/averages [Ave]).

#### Study Design and Participant Selection Criteria

Using a cross-sectional study design and convenience sampling technique, we considered 26 content experts, identified by colleagues or via online search and sent an introductory email to each of the 13 who met the inclusion criteria (minimum of 3 years of productive work involving the CHWs, Master's degree, and at least three relevant CHW performance-related publications within the South African context).

#### Data Collection/Pilot Testing

Via email, we sent the following: participants' information sheet, the quality-of-care assessment tool, and the fieldwork manual guiding the use of the tool. We developed a validation questionnaire including questions on various items in the tool (with a series of ratings on a Likert scale). We provided space for additional comments including missing items from the tool or any other comments. Each expert reviewer worked independently and anonymously. We also offered the experts the option of a meeting (virtually) to complete the questionnaire. We collected data between 15 July 2020 and 28 August 2020.

We had the questionnaire piloted by three researchers to assess clarity, flow, comprehension, and grammar. We used their comments and suggestions to strengthen the questionnaire.

#### Data Analysis

We computed the CVI by asking experts to rate each item relevance on a scale of five (1=*strongly disagree*; 2= *somewhat disagree*; 3= *neither agree nor disagree*; 4= *somewhat agree*; and 5= *strongly agree*). Then we categorized ratings of 4 or 5 as ‘1=relevant' and one–three as ‘0 =not relevant'. To calculate the I-CVI, if there are five or fewer experts, all must agree (that is all must have assigned a score of either four or five with an overall I-CVI of 1.0). For six or more experts, calculated I-CVI must be ≥0.83 for the item to be content valid, that is, not all the experts need to agree (46). The I-CVI is further categorized as follows: <70% (to be eliminated), 70–79% (needing revision), and >79% (appropriate) (48). Therefore, based on the ratings of this study's six experts, we calculated I-CVI for each item by dividing the number of experts whose scores were categorized as ‘1' by the number of experts who provided a score at all for each item.

To calculate S-CVI, two methods are used: (1) S-CVI/UA assesses in how many items in the tool, overall, was there universal agreement (UA) based on the experts' scores, or (2) S-CVI/Ave which assesses the average (Ave) of all individual item index (I-CVIs) derived from experts' scores (50). To calculate S-CVI/UA, we derived the proportion of items for which the experts scored as ‘1', divided by the total number of items in the tool. In the S-CVI/Ave approach, we summed up the I-CVIs for all the items and divided that by the total number of items in the tool (51, 52). As in previous study, to establish the relevancy of the overall items of our new tool, for both methods, the index had to be ≥80% (excellent) (53).

Thematic analysis was performed for qualitative data (experts' comments), driven by questions posed to the experts. The findings were summarized across all the experts to identify what was common or different, that are thought to improve the tool.

### Inter-rater Reliability

For the IRR, we used the following methods: (i) Bland and Altman (54) recommend a widely-used method that takes into account sources of variations between measures to determine the degree of agreement, which should be predetermined by the researchers (55–57); (ii) For the analysis of clustered data (i.e., clustering of patients grouped within households and by site), we performed the multilevel model analysis, which considers correlations among responses of observed units within clusters (58) to obtain statistically efficient estimates of the regression coefficients of quality-of-care messages or communication scores on observer and site.

#### Study Design

This study used the secondary data collected during the evaluation phase (endline) of a 3-year (2016–2019) intervention study (Batlhokomedi project) (33, 34). Details of the primary data collection and study design have been reported elsewhere (34). Briefly, a mixed method design was used, which included a cross-sectional observational study of randomly selected CHWs analyzed in this study.

#### Data Collection

Briefly, in the primary study, data were obtained from 110 households in Area A and 106 households in Area B of Gauteng Province, South Africa. Furthermore, in 21 household visits where two observers observed the same CHW and scored the visits using the quality-of-care assessment tool, we obtained complete data from 18 (9 per area) households. All data were received in Excel spreadsheet.

#### Data Analysis

To determine the degree of agreement, difference in the mean data were plotted against the mean and an equality line on which all the points would lie if the measures were the same reading each time was drawn (Bland-Altman plot). The degree of agreement, or the lack was then determined by calculating the bias (limit of agreement) from the mean difference (*d*) and the standard deviation (SD) of the differences (s), assuming that the difference is normally distributed. That is:


$$\begin{array}{l} \text{Limits of agreement }(\text{LOA})\;=\text{mean difference }\pm 1\;.96\\ \;\times \;\left(\text{SD of differences}\right) \end{array}$$

For the Bland Altman analysis, we included all paired observer data (21 household records [area a, *n* = 11; area b, *n* = 10]). We derived the quality-of-care messages or communication scores as earlier described. After excluding households with missing quality-of-care messages or communication scores, we included nine pairs of household observations per site in the analyses. The dependent variables were quality-of-care messages or communication score. In this analysis, we considered observer and site as fixed effects (that is, the levels of these factors are selected by a non-random process or consist of the entire population of possible levels; for example, “area” [1 = a, 2 = b] is fixed since there are only two possible values, both of which are included in our model). We also performed a fixed effect multilevel modeling including ‘area' and ‘observer' to calculate the proportion of the total variance that is between observers (or due to observers) as:


$$\begin{array}{l} \rho \;=\;{\sigma \text{u}}^{2}/{\sigma \text{u}}^{2}\;+\;{\sigma \text{e}}^{2} \end{array}$$

where ρ refers to the reliability or the intra-class correlation that measures the closeness of scores assigned by the same observer relative to the closeness of scores by a different observer.

#### Ethics Approval and Consent to Participate

This study was approved by the Human Research Ethics Committee (Medical) of the University of the Witwatersrand, Johannesburg (approval number M190933). For the face validity, we also obtained ethical approval from Ehlanzeni (MP-2020001-002) and Tshwane Districts (GP-202001-012). We obtained written informed consents from all face validity participants. For the experts, completion of the validation questionnaire was synonymous with consent.

## Results

### Face Validity

In this study, we included data of participants from facilities A and B in Mpumalanga Province and C and D in Gauteng Province. From facilities A, B, C and D; 5, 6, 5, and 5 CHWs; and 1, 2, 0, and 3 OTLs, respectively, provided ratings on our tool (Table 3).

**Table 3:** Percentage agreement of ratings by CHWs, OTLs, province and overall.

**Variable**	**Extent of agreement on content (tool covers everything done during household visits)**		**Extent of agreement on acceptability (happy to be assessed using the tool)**	
	**Mean score**	**Agreement index (%)**	**Mean score**	**Agreement index**
**CHWs**				
Facility				
A (*n =* 5)	5.00	100.00	5.00	100.00
B (*n =* 6)	4.80	96.00	4.80	96.00
C (*n =* 5)	4.40	88.00	3.20	64.00
D (*n =* 5)	5.00	100.00	4.40	88.00
Province				
MP (*n =* 11)	4.90	98.00	4.90	100.00
Gau (*n =* 10)	4.70	94.00	3.80	76.00
Total (*n =* 21)	4.80	96.00	4.35	87.00
**OTLs**				
Facility				
A (*n =* 1)	5.00	100.00	5.00	100.00
B (*n =* 2)	4.50	90.00	4.00	80.00
C (*n =* 0)	0.00	0.00	0.00	0.00
D (*n =* 3)	4.67	93.30	4.67	93.30
Province				
MP (*n =* 3)	4.75	95.00	4.50	90.00
Gau (*n =* 3)	4.67	93.30	4.67	93.30
Total (*n =* 6)	4.71	94.20	4.59	91.70

*CHWs, Community health workers; OTLs, Outreach team leaders; MP, Mpumalanga (Facility A and B); Gau, Gauteng (Facility C and D)*.

The mean years of experience were 15.0, 8.1, 9.0, and 9.7 years for CHWs and 2.4, 2.5, no data, and 4.5 years for OTLs, respectively. Among CHWs, only those in facility B had passed the Ward-based Outreach Team Phases one and two examinations. Only one CHW in facility A passed the Phase one examination.

All the CHWs and their leaders agreed that the assessment tool covered everything that happens during their household visits (with overall mean scores and agreement index of 4.8 vs. 4.7 corresponding to 96.0 vs. 94.2% agreement index, respectively) (Table 3). Although they also agreed that they were happy to be assessed using the tool overall (4.35 vs. 4.59 corresponding to 87.0 vs. 91.7%, respectively), the mean score and index for CHWs in facility C was 3.2 and 64.0%, respectively. In facility C, of the five respondents, only one gave a rating of four, regarding whether they were happy to be assessed using the tool. Qualitatively, one OTL indicated that the fieldworker rating (global rating) for the CHWs should be objective.

### Content Validation

Of the 13 eligible experts invited to this study, six (response rate, 46%) agreed to participate and provided both qualitative and quantitative judgements on the tool items.

The I-CVI of the 11 items ranged between 0.83 and 1.00. For three items (“assessment of quality of communication,” “assessment of messages and activities,” and “global rating”) fewer than six experts assigned a score of four or five. However, the I-CVI proportion was above 0.8 for each of these three items. Therefore, having all the 11 items in the tool is appropriate.

For the S-CVI/UA, the six experts assigned a score of 4 (quite relevant) or 5 (very relevant) for 8 of 11 items. Therefore, the S-CVI/UA was 0.72. With S-CVI/Ave, the average of the proportions (or I-CVIs) where all the experts had rated the items as relevant was 0.95.

For *item 7*, checklist aggregate score (that is, the quality-of-care messages score)' *and eight ‘*global ratings,' only one of six experts rated that a CHW with high scores would not be a genuinely high-performing CHW (or vice versa). However, on *item 7*, three experts commented as follows:

“*There is a limit to the extent to which a score can capture the relational nature of communication during a visit*”–Expert 1“*Collaboration and cooperation need attention*”–Expert 2“*Data must be triangulated from observation, the CHWs documentation, performance reviews, and/or household member experience*”–Expert 3

On *item 8*, three experts commented that the global ratings (*item 8*), are subjective. Even the remaining three suggested the need for triangulation with other aspects of data (CHW documentation or household member experience) for appropriate interpretations. Three of four experts who provided comments on communication (*item 2*) suggested that other aspects of communication including listening skill (i.e., body language, respect and empathy, rapport, and rapport-related skills such as praise and affirmation), be added to this tool. On notetaking, two of three experts considered the relevance of electronic forms of notetaking, while another suggested the need to also consider post-visit notes.

### Inter-rater Reliability

Table 4 shows the mean SD of quality-of-care messages and communication scores per paired observers, by site, and households. The mean messages scores differed between observers by household and site.

**Table 4:** Quality-of-care mean messages and communication scores difference by paired observers by site.

	**Mean score difference between observers**			
	**Site a**		**Site b**	
	**Observer (a) vs. (b)**	**Observer (a) vs. (c)**	**Observer (a) vs. (b)**	**Observer (a) vs. (c)**
**N households per observer**	**5**	**4**	**8**	**1**
**Quality-of-care assessment tool messages score (household level)** ^a^	0.17 (range, 0.72–0.89)^b^	0.05 (range, 0.53–0.58)^b^	0.11 (range, 0.66–0.77)^b^	0.26 (range, 0.52–0.78)^b^
**Quality-of-care assessment tool communication score (household level)** ^a^	0.00 (range, 1.00–1.00)	0.07 (range, 0.93–1.00)	0.11 (range, 0.89–1.00)	0.50 (range, 0.50–1.00)

a *Observer a consistently had the highest scores across all the paired categories;*

b *Range shows the scores per paired observers*.

**Figure 1:**
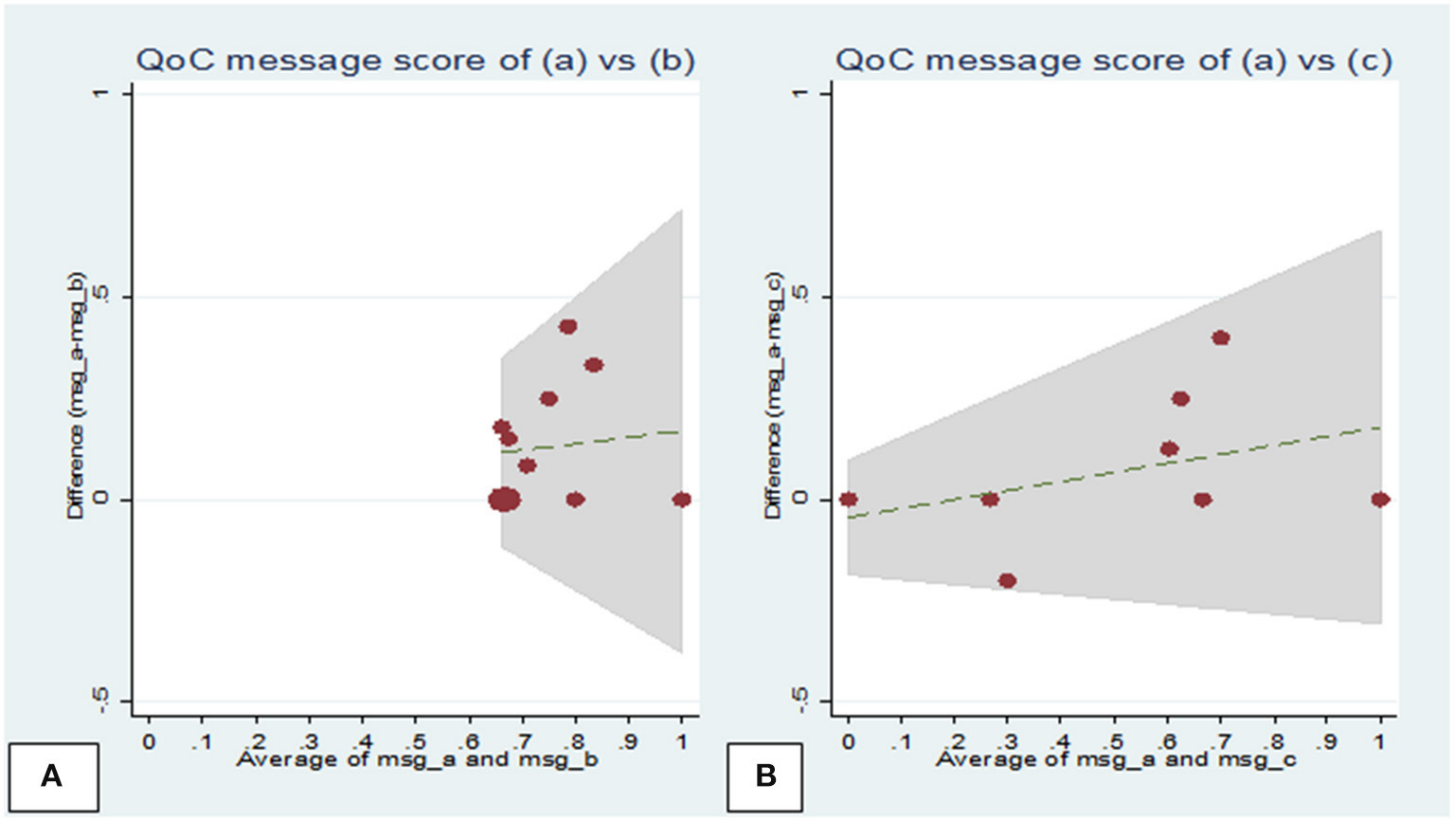
Bland-Altman plots of the mean difference against the mean quality-of-care messages scores assigned by observers during household visits. **(A)** Plot showing observers (a) and (b) scores. Mean difference = 0.14, standard deviation (SD) = 0.20 (limits of agreement = mean−2 × SD to mean+2 × SD) = −0.18 to 0.44; **(B)** Plot showing observers (a) and (c) scores. Mean difference =0.07, SD = 0.18 (limits of agreement = mean−2 × SD to mean+2 × SD) = −0.30 to 0.44. QoC, quality-of-care; msg, message.

**Figure 2:**
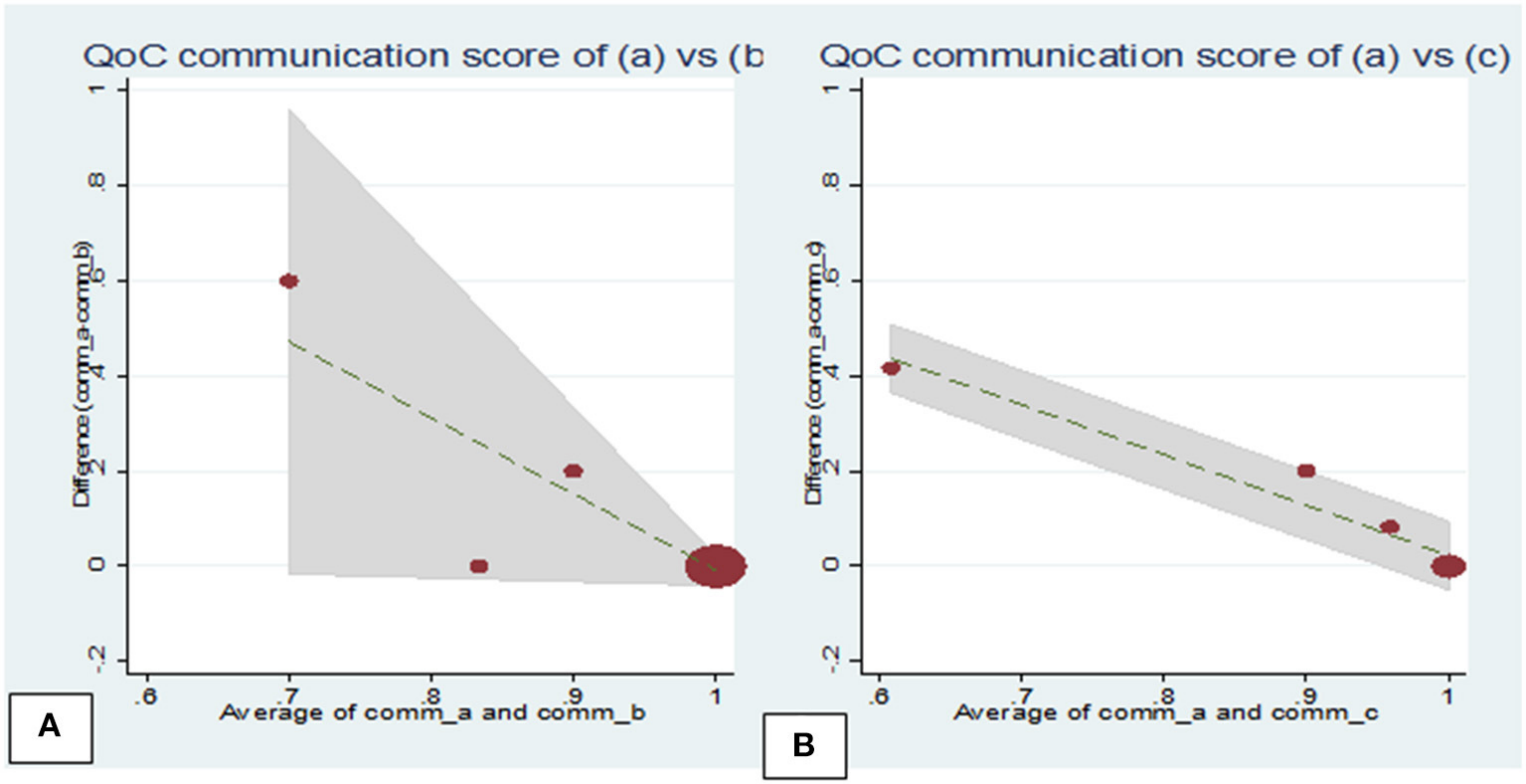
Bland-Altman plots of the mean difference against the mean communication scores assigned by observers during household visits**. (A)** Plot showing observers (a) and (b) scores, mean diff = 0.07, standard deviation (SD) = 0.19 (limits of agreement = mean−2 × SD to mean+2 × SD) = −0.31 to 0.44; **(B)** Plot showing observers (a) and (c) scores, mean diff = 0.14, SD = 0.20 (limits of agreement = mean−2 × SD to mean+2 × SD) = −0.25 to 0.54.

The Bland-Altman plot for the quality-of-care messages scores revealed that the LOA ranged from −0.18 to 0.44 [between observers (a) and (b) (Figure 1A)] and from −0.30 to 0.44 [observers (a) and (c) (Figure 1B)]; while for the quality-of-care communication scores, these ranged from −0.22 to 0.45 [observers 1 and 2 (Figure 2A)] and from −0.28 to 0.40 [observers 1 and 3 (Figure 2B)]

The multilevel modeling revealed an estimated reliability of 0.77 for observations between different observers in the same household compared to observations on different households for the quality-of-care messages scores, and 0.14 for the quality-of-care communication scores (Table 5).

**Table 5:** Estimated inter-rater reliability in multilevel modeling analysis.

	**Reliability between raters in the assigned quality-of-care messages scores**	**Reliability between raters in the assigned quality-of-care communication scores**
Sigma u (σu)	0.21	0.05
Sigma e (σe)	0.12	0.12
Rho (ρ) = σu^2^/σu^2^ + σe^2^ =inter-rater reliability	= 0.21^2^/(0.21^2^ + 0.12^2^) =0.77	=0.05^2^/(0.05^2^ + 0.12^2^) =0.14

## Discussion

The results of this study suggest that the quality-of-care assessment tool has a high face and content validity, and the IRR was substantial for quality-of-care messages but poor for communication scores. The low IRR of 14% for quality-of-care communication scores suggest that observer differences were important. Discrepancies in scores have been related to personal bias in the scoring process and skill deficiencies especially among newly trained observers (59, 60). The communication items also, may be more subjective than the messages scores that are in line with the South African CHW training manual (39). This is because communication could be affected by stress, emotion and workload and may require additional training of the observers on speech recognition (61). These results suggest that the quality-of-care messages score may be useful summative assessment, but this would provide a partial assessment. The communication scores, and perhaps the whole tool, may be best used to assess CHW activities only formatively. That is, the assessment may only provide the basis for reflecting and discussing about CHW performance in order that these might lead to change.

From a theoretical perspective, formative assessment provides an interphase between social interaction (teacher-learner) and cognition (learning) through which learners' thinking and learning processes are supported (62). Through our quality-of-care score, CHW independent work during household visits can be assessed on an ongoing basis, with timely reflective information and feedbacks on learning gaps to help improve independent CHW care and comprehensive care outcomes (63). The CHWs learning process as well as mentorship/supervision approaches can be enhanced through feedbacks, self-reflection, and dialogue (64). Abundance evidence exists on the relevance of formative assessment in the learning process of healthcare trainees including undergraduate, postgraduate, and nursing education globally, for required skills (65–68).

In low-and-middle-income countries, formative assessment approaches have been examined in healthcare workers. In Malawi, the effect of a formative assessment framework among nursing studies in the teaching and learning of essential nursing skills resulted in improved competencies in the skills laboratory (69). Intensive care skills training of intensive care unit nurses including a formative assessment component, was effective in improving participants' knowledge on assessment and management of patients (70). To become a CHW, the required educational qualification is minimal (12). However, despite this limited educational background, CHWs are required to work within complex inter-relating environments of community and health sector within which are multiple layers of actors whose actions have effect on CHW performance (71). Therefore, as Agarwal et al. suggested, a formative assessment approach, measured routinely, built into supervisory activities, using a checklist, may provide opportunity for immediate and comprehensive feedback (17). Such practices when carried out in an environment that nurtures the development of CHW learning, by providing allowance for making mistakes and rectifying them without compromising patients care, would be appropriate for CHWs (72). Our tool can be applied in CHWs work environment, before and after regimented training, and on an ongoing basis, with non-judgmental feedbacks on activities requiring strengthening, by healthcare system actors including OTLs, managers, and supervisors to enhance CHW skills.

Recently, focus is shifting to peer formative assessment as another approach to enhance student engagement with learning (73) improve teamwork skills (74) and provides multiple opportunities for assessment of competence by peers (75). In an intervention study of lady health workers in Pakistan, CHWs who received additional 4-day clinical and supervisory training provided supportive supervision to their peers during household visits (76). A component of the intervention required the peer supervisors to provide written feedback to their peers. The group who received this feedback showed better improvement. This underscores the relevance of a formative assessment tool not only for supervisors and team leaders, but also peers.

### Strength and Limitation

We developed a tool that might be applicable for formative assessment in programs where the CHWs provide comprehensive care, to strengthen individual CHW learning through reflective feedback. However, the study has a few limitations. (1) A low face validity was reported in one of four facilities included in the face validity study. This was the only facility where the researcher, instead of the OTL, was left to approach and secure individual CHW buy-in. Challenges with inexperienced OTL, considered in this instance, include poor communication and problems with managing team members. On the other hand, the self-selected CHWs may be a more representative sample than a possible ‘preselected-to-provide-positive-feedback' group. However, despite this facility's low validity, the comments section of CHWs ratings by those who would not be happy to be assessed using this tool offered no information on why they would not be happy. (2) Assessments and comments by experts may have emphasized their specialty more, as implied by lower ratings on some items. Thus, few experts whose most dominant expertise was on curriculum development and communication required further clarifications before being able to provide a rating on items in their less-dominant areas, with the tendency for more cautious ratings on such items. (3) Our IRR assessment data had a small sample size due to logistic issue. A larger multi-country study could provide findings to enhance generalizability across wider national CHW programs.

## Conclusion

We provide a simple tool to facilitate the provision of feedback to strengthen individual CHW activities with a view to progressive improvement in the levels of competence. In our future study, we hope to explore the integration of our tool with continuing education and supportive supervision for CHW work. The use of our tool can be encouraged by policy makers and actors within the healthcare system to improve CHW practice, especially for comprehensive care, and within national CHW programs.

## Data Availability Statement

The original contributions presented in the study are included in the article/Supplementary Material, further inquiries can be directed to the corresponding author/s.

## Ethics Statement

The studies involving human participants were reviewed and approved by the Human Research Ethics Committee (Medical) of the University of the Witwatersrand, Johannesburg (approval number M190933). Ehlanzeni (MP-2020001-002) and Tshwane Districts Research Committees (GP-202001-012). The patients/participants provided their written informed consent to participate in this study.

## Author Contributions

OB, JG, and FG conceptualized the study and raised the funding. OB led the drafting of the manuscript and was responsible for overseeing data collection. OB, FG, JL, and CB were responsible for the data analysis. OB, JG, FG, CB, and JL contributed to drafting the manuscript. All authors contributed to the article and approved the submitted version.

## Funding

The study was funded by Medical Research Council UK, DFID, ESRC and Wellcome Trust under the Joint Health Systems Research Initiative (grant number, MR/N015908/1) and by the South African Research Chairs Initiative (SARChI) (grant number, 87369). CB is supported by the National Institute for Health and Care Research (NIHR) Applied Research Collaboration (ARC) West Midlands. The views expressed are those of the author(s) and not necessarily those of the NIHR or the Department of Health and Social Care.

## Conflict of Interest

The authors declare that the research was conducted in the absence of any commercial or financial relationships that could be construed as a potential conflict of interest.

## Publisher's Note

All claims expressed in this article are solely those of the authors and do not necessarily represent those of their affiliated organizations, or those of the publisher, the editors and the reviewers. Any product that may be evaluated in this article, or claim that may be made by its manufacturer, is not guaranteed or endorsed by the publisher.

## Abbreviations

Ave: average
CHW: community health worker
c-IMCI: community-based integrated management of childhood illnesses
CVI: content validity index
Gau: Gauteng Province
HCSI: Home Visit Communication Skills Inventory
I-CVI: item-level CVI
IRR: inter-rater reliability
LOA: limit of agreement
mMRIGs: mobile media-rich interactive guidelines
MP: Mpumalanga Province
OTL: outreach team leader
QoC: quality-of-care
S-CVI: scale-level CVI
SD: standard deviation
TB: tuberculosis
UA: universal agreement.
