# Supplementary material for: Assessing the Utility of a Quality-of-Care Assessment Tool Used in Assessing Comprehensive Care Services Provided by Community Health Workers in South Africa
Source: Front Public Health. 2022 May 16;10:868252. doi: 10.3389/fpubh.2022.868252 (PMC9149253; doi:10.3389/fpubh.2022.868252)
Supplement: Supplementary file 1 [file Table_1.docx]

Supplementary Material

# Supplementary Tables

**Supplementary Table 1 Results of scoping review for studies evaluating tools assessing community health worker performance in LMICs**

| **CHW Performance Measurement Framework indicator for CHW competency**^a^ | **Previous study author and year** | **Scope of CHW work (single disease/condition or comprehensive care)** | **Performance measure** | **How was the indicator measured (assessment methods)?** | **Type of tool designed for data collection (assessment tools)** | **Type of utility reported for the assessment tools** |
| --- | --- | --- | --- | --- | --- | --- |
| *-*CHW knowledge | Bailey 1996[1] | Diarrhoea case management | Knowledge score | Case-based pre-test and post-test before and after training | Written/oral examination | None |
|  | Gautham et al. 2015[2] | Integrated Management of Childhood Illnesses (IMCI) | Pre- and post-training test score | Assessment of pre- and post-training were based on | Simple assessment instrument | None |
| -Service delivery/quality | Ayele et al. 1993[3] | All activities specified in the job description for community health agents (any CHW included in the study was required to be performing at least one activity) | Completion of activities by CHWs as set by the government was used to derive a functional status score. | Direct observation by supervisor at onset, three and six months. | Checklist | None |
|  | Agarwal, Gallo, Finlay 2013[4] | Community‐based Integrated Management of Childhood Illness (c‐IMCI) | The mean percentage of correctly performed activities was calculated as the Community‐based Integrated Management of Childhood Illness (c‐IMCI) performance score (score 0-100) | Direct observation by trained experts | Expert observers scored the  encounters using a standard observation checklist. | Proportion of recommended  treatments that were prescribed correctly by CHWs were compared to gold standard |
|  | Ashraf et al. 2015[5] | Household visits for environmental inspections, counselling on  women’s and children’s health, and referrals | The number of household visits completed over the study period. | Administrative data | Signed receipts per household visited | Validation via household report of CHW activity and comparison to HMIS administrative data |
|  | Gautham et al. 2015[2] | Integrated Management of Childhood Illnesses (IMCI) | Protocol compliance following training on guideline-based care in use of mHealth system | Trained field investigators observed the CHW care to adult/paediatric patients | Media-rich, mobile  phone–based clinical guidance system for managing fevers, diarrhoeas and respiratory problems by rural health provider–mobile media-rich interactive guidelines (mMRIGs) | Two validated clinical guidelines were adapted and deployed in cell phones |
|  | Andreoni 2016[6] | Polio vaccination drives | Closeness to vaccine distribution policy objective | By smartphone application | CHW self-completed a vaccination monitoring application on a smartphone | None |
|  | Bossuroy et al. 2016[7] | TB case detection | TB case detection rate | Administrative data | Treatment card tracker was completed by survey staff to document patient-related treatment data using information from CHW Treatment card | Tracker card data were compared against government records |
|  | Taylor et al. 2018[8] | Case management of malaria, diarrhoea, pneumonia, pregnancy and newborn care, family planning and under-nutrition. | Relationship between selection scores and on-the-job performance | Data collected via phone-based application | Written tests and one-to-one interview | Predictive reliability |
|  | Laurenzi et al. 2020[9] | Maternal and  infant wellbeing, child nutrition, immunization, HIV/AIDS prevention  and treatment, and access to social and health services. | Home Visit Communication Skills Inventory (HCSI) score | The HCSI’s 21 items  covering active listening, active delivery, and active connecting domains,  was used to score English transcripts of audio‐recorded home visits interactions between CHWs and their maternal care clients. | Home Visit Communication Skills Inventory, a 21-item checklist | Inter-rater reliability |
| -Data reporting* |  |  |  |  |  |  |

LMIC, low and middle-income countries; CHW, community health workers; TB, tuberculosis

Notes: The data reported in Supplementary Table 1 were obtained through search in PubMed till 28 Feb 2020. *No study assessed data reporting a factor alone. ^a^Agarwal et al. (2019)[10]

**Supplementary references**

1. Bailey JE, Coombs DW. Effectiveness of an Indonesian model for rapid training of Guatemalan health workers in diarrhea case management. J Community Health (996) 21:269-76.

2. Gautham M, Iyengar MS, Johnson CW. Mobile phone–based clinical guidance for rural health providers in India. Health Informatics J (2015) 21:253-66.

3. Ayele F, Desta A, Larson C. The functional status of community health agents: a trial of refresher courses and regular supervision. Health Policy Plan (993) 8:379‐84.

4. Evaluation of the quality of community based integrated management of childhood illness and reproductive health programs in Madagascar [https://www.urc-chs.com/sites/default/files/Madagascar_Eval_qual_comm_IMCI_CDC_Feb2013.pdf]. Accessed 28 Feb 2020.

5. Ashraf N, Bandiera O, Lee SS. Do-gooders and go-getters: career incentives, selection, and performance in public service delivery: Citeseer. STICERD, LSE; 2014 Jul 27.

6. Andreoni J, Callen M, Khan Y, Jaffar K, Sprenger C. Using preference estimates to customize incentives: An application to polio vaccination drives in pakistan. In.: National Bureau of Economic Research (2016).

7. Bossuroy T, Delavallade C, Pons V. Fighting tuberculosis through community based counsellors: a randomized evaluation of performance based incentives in India. New Delhi: International Initiative for Impact Evaluation. 2016.

8. Laurenzi CA, Gordon S, Skeen S, Coetzee BJ, Bishop J, Chademana E, et al. The home visit communication skills inventory: Piloting a tool to measure community health worker fidelity to training in rural South Africa. Res Nurs Health (2020) 43:122-33.

9. 83. Taylor CA, Lilford RJ, Wroe E, Griffiths F, Ngechu R. The predictive validity of the Living Goods selection tools for community health workers in Kenya: cohort study. BMC Health Serv Res (2018) 18:803.

10. Agarwal S, Sripad P, Johnson C, Kirk K, Bellows B, Ana J, Blaser V, Kumar MB, Buchholz K, Casseus A et al. A conceptual framework for measuring community health workforce performance within primary health care systems. Hum Resour Health (2019) 17:86.
